# Psychometric Evaluation of the Czech Version of Group Cohesiveness Scale (GCS) in a Clinical Sample: A Two-Dimensional Model

**DOI:** 10.3389/fpsyg.2020.595651

**Published:** 2020-12-03

**Authors:** Adam Klocek, Tomáš Řiháček, Hynek Cígler

**Affiliations:** Department of Psychology, Faculty of Social Studies, Brno, Czechia

**Keywords:** confirmatory factor analysis, group cohesion, Group Cohesiveness Scale, Czech validation study, affective and behavioral group cohesion

## Abstract

The Group Cohesiveness Scale (GCS, 7 items) measures patient-rated group cohesiveness. The English version of the scale has demonstrated good psychometric properties. This study describes the validation of the Czech version of the GCS. A total of 369 patients participated in the study. Unlike the original study, the ordinal confirmatory factor analysis (CFA) supported a two-dimensional solution (RMSEA = 0.075; TLI = 0.986). The analysis demonstrated the existence of two moderately to highly associated (*r* = 0.79) domains of group cohesiveness—affective and behavioral. The two-dimensional model was invariant across genders, age, education, and time (retest after 6 weeks) up to factor means level. Internal consistency reached satisfactory values for both domains (affective, ω = 0.86; behavioral, ω = 0.81). In terms of convergent validity, only weak association was found between the GCS domains and the group working alliance measured by the Group Outcome Rating Scale (GSRS). This is the first revision of the factor structure of the GCS in the European context. The scale showed that the Czech version of the GCS is a valid and reliable brief tool for measuring both aspects of group cohesiveness.

## Introduction

Group cohesion is one of the elemental group phenomena that allows other therapeutic processes to occur within the group therapy framework. It is defined as the ability of the members of a group to tolerate negative emotions and self-disclosure (Wongpakaran et al., 2013). Group cohesion partially overlaps with other group phenomena, such as the working alliance and empathy (Johnson et al., 2005). Group cohesion is conceptually akin to the working alliance in individual therapy. Although it is primarily based on the relationships among the group members, it can also be extended to the relationship with the therapists (Budman et al., 1989). Group cohesion is also related to empathy because a cohesive group demands that its members have an understanding of others’ feelings and experiences and can effectively express this understanding (Roark and Sharah, 1989).

Until recently, group phenomena and processes were measured by measures such as the Group Climate Questionnaire (MacKenzie, 1983), the Therapeutic Factors Inventory (Lese and MacNair-Semands, 2000), and the Working Alliance Inventory (Horvath and Greenberg, 1989). However, these scales were too lengthy to be used in routine care or rapid hospital environments (compared to research) and were not directly focused on group cohesion. Therefore, the Group Cohesiveness Scale (GCS^1^) was developed (Wongpakaran et al., 2013).

The GCS (Wongpakaran et al., 2013; see Table 1) was created from an original pool of 40 items and reduced to seven items representing two domains: cohesion and engagement. The former domain was represented by two items from the Therapeutic Factors Inventory, while the latter was represented by five items from the Group Climate Questionnaire. However, since both domains were similar in content, Wongpakaran et al. (2013) considered them to be representations of the unidimensional group cohesiveness construct.

**TABLE 1:** Group Cohesiveness Scale (Wongpakaran et al., 2013).

**Item no.**	**Item wording (*Czech in italics*)**	**Cohesiveness (C) or engagement (E) domain**	**Affective (A) or behavioral (B) domain**
1	I feel accepted by the group. (*Cítím se být skupinou přijímaný/á*.)	C	A
2	In my group, we trust each other. (*Ve skupině si vzájemně důvěřujeme*.)	C	A
3	The members like and care about each other. (*Členové skupiny se mají rádi a vzájemně jim na sobě záleží*.)	E	A
4	The members try to understand why they do the things they do; they try to reason it out. (*Členové se snaží porozumět tomu, proč dělají věci, které dělají; snaží se na to přijít*.)	E	B
5	The members feel a sense of participation. (*Členové skupiny cítí, že se podílejí na chodu skupiny*.)	E	B
6	The members appear to do things the way they think will be acceptable to the group. (*Vypadá to, že členové dělají věci způsobem, o němž si myslí, že bude pro skupinu přijatelný*.)	E	B
7	The members reveal sensitive personal information or feelings. (*Členové si sdělují citlivé osobní informace a pocity*.)	E	B

Alternatively, Wongpakaran et al. (2013) suggested that the GCS items can be differentiated into the affective (items 1, 2, and 3) and behavioral (items 4, 5, 6, and 7) components of group cohesiveness. They argued that these components might be related to each other in a fashion similar to the unidimensional construct of depression, in which the feeling of sadness is functionally different from a behavioral lack of interest, yet both components measure the same latent construct of depression (Wongpakaran et al., 2013).

The distinction between the affective and behavioral components is consistent with the theoretical literature. According to Carron (1982), group cohesion is a “dynamic process that is reflected in the tendency for a group *to stick together* [emphasis added] and remain united in the *pursuit of its goals and objectives.* [emphasis added]” (p. 124). Similarly, Mudrack (1989) divided group cohesion into attraction-to-group (affective component) and commitment to the group task (behavioral component).

Originally, the GCS was standardized in the Thai language (Wongpakaran et al., 2013) in a clinical sample of 96 patients (56% women) with a mean age of 28.22 (*SD* = 6.84). Patients were hospitalized for up to 2 weeks. A principal component analysis revealed a unidimensional factor structure (57.2% of explained variance). Based on a confirmatory factor analysis (CFA) conducted on the same dataset, the authors claimed that the unidimensional model had moderately acceptable fit despite unsatisfactory RMSEA values (χ^2^(14) = 32.29; CFI = 0.94; TLI = 0.90; SRMR = 0.04; RMSEA = 0.12).

Although Wongpakaran et al. (2013) tried to fit a two-dimensional model (i.e., cohesion and engagement), they did not report the results, arguing that the two dimensions were too strongly correlated to be set apart (*r* = 0.83). Instead, they fine-tuned the unidimensional model based on modification indices by allowing residual correlations between pairs of items (items 1 and 2; items 2 and 3), reaching an excellent fit [χ^2^(12) = 12.41; CFI = 0.99; TLI = 0.99; SRMR = 0.04; RMSEA = 0.02]. Arguably, by allowing the residual correlations, the authors developed a model that was very similar to (but less parsimonious than) the suggested two-factor model with the affective and behavioral factors. Therefore, we found it desirable to formally test this alternative two-factor model as well. In terms of convergent validity, the GCS was correlated to the Group Benefit Questionnaire (*r* = 0.71, *p* < 0.001) and to the Cohesion to Therapist Scale (*r* = 0.77, *p* < 0.001) in the original study.

The GCS is a relatively new measure that has been employed in a limited number of studies thus far. Psychometric information about the GCS is rather scarce and often unsatisfactory given small sample sizes. Poyner-Del Vento et al. (2018) used the GCS as a measure of group cohesion in a pilot study in a sample of seven female military veterans. They found that removing item 6 (“*The members appear to do things the way they think will be acceptable to the group*”) increased the internal consistency of the scale from α = 0.72–0.90. Tulin et al. (2018) used the GCS to measure group cohesion in a sample of 109 students with internal consistency of α = 0.90. In another sample of 22 students, Ashby et al. (2018) found a mean interitem correlation of *r* = 0.43. This limited evidence does not allow us to thoroughly evaluate the GCS, and the applicability of the measure in Western culture is still missing.

This study aimed to validate the Czech version of the GCS using the ordinal CFA paradigm. Four models were tested, including the unidimensional model (model 1), the unidimensional model with residual covariances between items 1 and 2 and items 2 and 3 allowed (model 2), a two-factor model with the factors of cohesion (items 1 and 2 originally extracted from the Therapeutic Factors Inventory) and engagement (items 3–7 originally extracted from the Group Climate Questionnaire) (model 3), and a two-factor model with affective (items 1–3) and behavioral factors (items 4–7) (model 4). Furthermore, to assess the convergent validity, we used the Group Session Rating Scale (GSRS, Quirk et al., 2013), a measure of the group working alliance, as a comparison. Although group cohesion and group working alliance are distinct constructs, we expected the GCS scores to be related to the GSRS scores because both instruments measure non-specific group-based relational factors of the therapeutic process.

## Materials and Methods

### Sample and Procedure

The sample included patients from seven clinical sites in the Czech Republic who provided informed consent to participate in research tracking the mechanisms of change during psychotherapy from January 2018 to December 2019. All patients underwent group therapy lasting from 4 to 12 weeks (depending on the site, median of 6 weeks). Data were collected on a paper-and-pencil form on a weekly basis during the whole treatment. Participants completed a battery of questionnaires regarding demographic variables, several outcome variables and several mechanisms of change, including group cohesion and working alliance. The study was approved by the Research Ethics Committee of Masaryk University (Ref. No. EKV-2017-029-R1).

In this study, the dataset used to validate the GCS included data from the second week of therapy (i.e., the first measurement of the group cohesion). Out of 448 patients who provided their baseline data, 380 patients (85%) participated in the second week of treatment. Out of 380 participants, 11 were characterized by missing data regarding the GCS, resulting in a total sample size of *N* = 369 patients. Differences between participants with missing data (*n* = 80) and the final sample (*n* = 369) in the demographics and clinical diagnosis data were investigated using *t*-tests and χ^2^-tests.

### Group Therapy

The treatment was integrative with major psychodynamic and minor humanistic and experiential aspects, supplemented with art, physical activity, music, ergo-, drama-, physio-, and biblio-therapy, relaxation and cognitive training, and community meetings^2^. Five sites were characterized by a frequency of five sessions of psychotherapy per week. The remaining two sites had three and four sessions per week, respectively. A session of group therapy lasted 90 min^3^.

The sample comprised small closed groups of inpatients within four clinical sites and small open groups of outpatients in a program with a daycare basis within three clinical sites. Twenty-five (16 female) therapists participated in this research (*M*_age_ = 44.13 years, *SD*_age_ = 10.29). They were trained in the psychodynamic or psychoanalytic approach (*n* = 15), gestalt (*n* = 4), person-centered approach (*n* = 3), integrative approach (*n* = 2) or Daseinanalysis (*n* = 1). Their experience fluctuated between 1 and 25 years (*M* = 12.21, *SD* = 7.30).

### Instruments

#### Group Cohesiveness Scale (GCS)

The seven items of the GCS are scored on a Likert scale from 1 (strongly disagree) to 5 (strongly agree). None of the items is negatively worded. A higher score indicates higher perceived group cohesion. In the original study, the GCS yielded an average score of 4.73 out of 5 (*SD* = 0.62), the internal consistency of the whole scale was α = 0.87, and the item-total correlations ranged from 0.497 to 0.752.

The scale was translated into Czech from the English version. Five native Czech speakers (a psychology student, two psychologists, and two laypeople) created five independent Czech translations. A group of three people (the two psychologists and the psychology student) then discussed all the translations and consolidated them into a single version. Third, this version was back-translated into English by a bilingual, native English speaker and compared to the original English version. Fourth, the final Czech version was field-tested with five respondents to check the comprehensibility of the items.

#### Group Session Rating Scale (GSRS)

The GSRS (Quirk et al., 2013) is a measure of the working alliance in group psychotherapy. It includes four 10-cm-long visual analog scales, each framed by a verbal anchor on both ends. The continuous dimension of each item is framed by bipolar points, and participants rate the group working alliance by making a mark on each scale. The response is measured as the length of the line from the left-hand side to the mark in millimeters. The range of the total score, computed as the sum of all items, can thus reach values between 0 and 400. A higher score indicates a better perceived working alliance. The scale was reported to be unidimensional, and the internal consistency ranged from α = 0.86 to 0.90 in the original study.

### Data Analysis

#### Software and General Settings

The statistical procedures were performed using statistical software R, version 4.0.2 (R Core Team, 2020). The significance level was set at *p* < 0.05.

#### Factorial Validity

The factor structure was estimated through ordinal CFA using the lavaan package (Rosseel, 2012). The ordinal factor analysis is equivalent to the two-parameter logistic graded response model in item response theory. Hence, this approach is not as vulnerable to the violation of assumptions as the standard factor analysis (Raykov and Marcoulides, 2011). Each item has five parameters (one slope and four thresholds between all neighboring response options). All five models were estimated using the stochastic weighted least squares means and variance adjusted estimator method (WLSMV), which seems to perform well with ordered categorical data (Raykov and Marcoulides, 2011). The fit indices employed in this study included χ^2^, χ^2^/df, root mean square error of approximation (RMSEA), Tucker-Lewis index (TLI), comparative fit index (CFI), and standardized root mean residual (SRMR). According to Hu and Bentler’s (1999) and Hooper et al. (2008) evaluation criteria, the χ^2^/df should not exceed 3, the RMSEA should optimally be below 0.05, but values up to 0.10 are still considered to indicate a satisfactory fit. The SRMR should not exceed 0.08. Optimally, the TLI and CFI should be above 0.95; nevertheless, values above 0.90 are still considered to indicate a satisfactory fit.

Within models with more than one dimension (models 3 and 4), factors were allowed to be correlated. Since model 3 contained a factor represented only by two items, these items were constrained to load equally on their factor. Otherwise, models were identified by standardizing the latent variable. The internal consistency was estimated using bootstrapped Cronbach’s alpha and McDonald’s omega coefficients (McDonald, 1999). In terms of convergent validity, the association between the GCS and the GSRS was tested on the level of latent scores.

#### Measurement Invariance

The invariance was tested with regard to age, gender, education, and time. Measurement invariance was assessed by testing differences between nested models with continually increasing constraints: configural, metric (factor loadings), scalar (intercepts), strict (residuals), and factor means. Age groups were created by dividing the sample according to a median split. Gender invariance was assessed between male and female participants. Education invariance was assessed between higher (university, high technical school) and lower education (primary and secondary school with or without graduation) levels. Time invariance was assessed between the second and sixth weeks of group therapy (the sixth week was chosen pragmatically because in most sites, the therapy lasted only 6 weeks). We used four different fit indices to test the invariance, namely, Δχ^2^, ΔCFI, ΔSRMR, and ΔRMSEA. We employed “theta” parametrization and invariance guidelines with regard to ordinal data according to Wu and Estabrook (2016). Two groups are considered to be invariant if the item parameters (i.e., factor loadings, thresholds, intercepts, residuals, and factor means) are similar across groups.

Items 3 and 7 demonstrated missing response frequency at response option 1 (i.e., 1 or “strongly disagree”). The remaining items demonstrated near-to-missing response frequency (0.01) at response option 1. Response option 2 (unnamed) was also very seldom selected by the participants in all items. Therefore, all items were recoded into three categories (i.e., responses from 1 to 3 were recoded as a single category, representing a low level of group cohesion) for the purpose of testing the measurement invariance.

## Results

### Missing Data

No significant differences between the final sample (*N* = 369) and the respondents with missing responses or respondents not participating in the study at the second week (*n* = 80, who were the remaining part of the initial sample of 449 participants) were found for the mean age, gender, education, and psychiatric diagnosis. The pattern of missingness could be considered missing at random. Therefore, only complete cases were included in the analyses.

### Descriptive Characteristics

The total sample included 369 patients (73.7% females). Their nationality included Czech (95%), Slovak (2%), and others (3%). The patients’ ages ranged from 18 to 71 years (*M*_age_ = 39.6, *SD* = 11.1). Psychiatric diagnoses were represented as follows: F4x (*n* = 261), F3x (*n* = 69), F6x (*n* = 53), F5x (*n* = 8), and F1x (*n* = 7). Several participants possessed multiple diagnoses (n = 33), mainly a combination of F4x and F6x (*n* = 13), F3x and F4x (*n* = 9), and F3x and F6x (*n* = 7). The remaining demographic variables are reported in Table 2.

**TABLE 2:** Descriptive characteristics of the sample (*N* = 369).

**Variable**	**Categories**	***n***	**Percent**
Gender	Female	272	74%
	Male	90	24%
	Missing	7	2%
Household	In partnership	189	51%
	Single	71	19%
	With parents	39	11%
	Other	62	17%
	Missing	8	2%
Marital status	Single	178	48%
	Married	114	31%
	Divorced	67	18%
	Widowed	2	1%
	Missing	8	2%
Education	Primary school	17	5%
	Secondary school	180	49%
	High technical school	22	6%
	University	141	38%
	Missing	9	2%
Occupation	Employee	163	44%
	Unemployed	53	14%
	Invalidity pension	35	10%
	Entrepreneur	23	6%
	Student	20	6%
	Maternity leave	7	2%
	Retirement	4	1%
	Other	15	4%
	Missing	49	13%

The mean scores for each GCS item, the GCS total score, and the GSRS total score, as well as the internal consistency of the unidimensional model, are reported in Table 3. The average total score was 3.7 (*SD* = 0.69). Corrected item-total correlations ranged from 0.49 to 0.75.

**TABLE 3:** Descriptive characteristics of scales (*N* = 369).

**Item**	***M***	***SD***	**Range (min-max)**	**Skewness**	**Kurtosis**	**Corrected item-total correlation**	**Cronbach’s alpha if item deleted**
GCS 1	3.70	0.91	4(1−5)	–0.01	–0.64	0.63	0.85
GCS 2	3.72	0.94	4(1−5)	–0.04	–0.87	0.75	0.84
GCS 3	3.51	0.92	3(2−5)	0.21	–0.85	0.69	0.85
GCS 4	3.81	0.94	4(1−5)	–0.27	–0.64	0.69	0.85
GCS 5	3.68	0.86	4(1−5)	–0.01	–0.53	0.69	0.85
GCS 6	3.57	0.93	4(1−5)	0.00	–0.44	0.59	0.86
GCS 7	4.00	0.93	3(2−5)	–0.36	–0.97	0.49	0.87

	***M***	***SD***	**Range**	**Skewness**	**Kurtosis**	**McDonald’s omega**	**Cronbach’s alpha**

GCS total	25.99	4.82	22(13−35)	0.20	–0.83	0.91	0.87 [0.85–0.89]
GSRS	290.82	75.20	368(32−400)	–0.66	0.09	0.83	0.82 [0.79–0.85]

### Factor Structure

First, the assumptions of factor analysis were tested. The data did not show multivariate normality, and the standardized residuals were positively skewed. Homoscedasticity was not observed. After the preliminary data analyses, an ordinal factor analysis was employed to estimate the fit of the factor models using these skewed non-linear data. The RMSEA of the null model was 0.398. This value is above 0.148; thus, the TLI fit index could be interpreted (Kenny et al., 2015).

**FIGURE 1:**
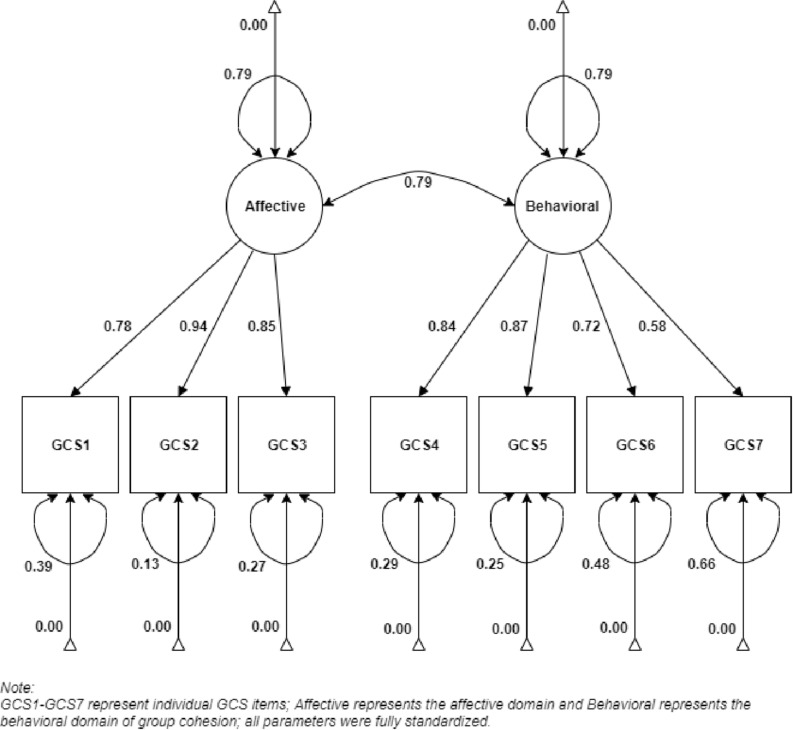
Factor structure of Group Cohesiveness Scale.

Second, four different factor solutions were tested for fit and compared (see Table 4). We concluded that the best fit was obtained by model 4, a two-factor solution with the affective and behavioral factors (see Table 5 and Figure 1). Model 4 fit the data significantly better than did model 1 [unidimensional; Δχ^2^(1) = 87.66, *p* < 0.0001] and model 3 [two-factor with the cohesion and engagement factors; Δχ^2^(2) = 104.31, *p* < 0.0001]. Furthermore, the fit of model 4 did not significantly differ from that of model 2 [unidimensional with residual correlations; Δχ^2^(1) = 2.35, *p* > 0.10]. However, model 4 can be considered superior in terms of parsimony as well as theoretical justification. While the affective factor represents the same underlying structure as the empirically derived residual correlations in model 2, it explains the item interrelationships more efficiently and is consistent with theoretical expectations (Carron, 1982; Mudrack, 1989).

**TABLE 4:** Fit indices of the tested models (*N* = 369).

**Model**	**χ^2^**	**df**	**χ^2^/df**	**TLI**	**CFI**	**SRMR**	**RMSEA**
**Model 1** (unidimensional)	249.67***	14	17.8	0.975	0.983	0.072	0.156 [0.133;0.180]
**Model 2** (unidimensional, Item 1 ∼∼ Item 2, Item 2 ∼∼ Item 3)	78.97***	12	6.6	0.994	0.997	0.041	0.076 [0.050;0.105]
**Model 3**ǂ (two-factor: cohesion and engagement)	238.95***	14	17.1	0.975	0.983	0.071	0.158 [0.135;0.182]
**Model 4**ǂǂ (two-factor: affective and behavioral)	79.71***	13	6.1	0.994	0.986	0.040	0.075 [0.049;0.102]

***p < 0.001.

*ǂCorrelation between the cohesion and engagement latent factors was r = 0.88.*

*ǂ ǂ Correlation between the affective and behavioral latent factors was r = 0.79.*

**TABLE 5:** Standardized regression weights (factor loadings) and errors (*N* = 369).

	**Model 1**		**Model 4**		
	**λ**	***h*^2^**	**λ_F__1_**	**λ_F__2_**	***h*^2^**
Item 1	0.75	0.56	0.78	–	0.61
Item 2	0.89	0.80	0.94	–	0.87
Item 3	0.82	0.67	0.85	–	0.73
Item 4	0.79	0.63	–	0.84	0.71
Item 5	0.83	0.69	–	0.87	0.75
Item 6	0.69	0.47	–	0.72	0.52
Item 7	0.56	0.31	–	0.58	0.34
*McDonald*’*s omega*	0.910		0.860	0.811	
*Raykov*’*s omega*	0.879		0.850	0.797	
*Cronbach*’*s alpha*	0.896		0.884	0.828	
*R*^2^	61.9%		32.7%	35.6%	

*R^2^, explained variance of the model;λ, factor loadings; h^2^, communality; F1, affective domain; F2, behavioral domain.*

*Correlation between F1 and F2 in model 4: r = 0.785. McDonald’s omega total for model 4 = 0.894.*

### Measurement Invariance

Measurement invariance was assessed for model 4 with respect to age, gender, and education (see Table 6). Several patients were lost due to missing responses on the demographic variables, namely, age (*n* = 9), gender (*n* = 7), and education (*n* = 9). Measurement invariance between the younger (*n* = 185) and older (*n* = 175) cohorts was reached on the configural, metric, scalar, factor mean, and residual levels. Even though the χ^2^-test was significant on the scalar and residual invariance level, other Δfit indices showed desirable values. Measurement invariance between women (*n* = 90) and men (*n* = 272) was reached on the configural, metric, scalar, and factor mean levels. Genders were not invariant only on the level of residual variances. Measurement invariance between lower (*n* = 197) and higher education levels (*n* = 163) was reached on the configural level. Even though the χ^2^-test was significant on both metric and scalar invariance levels, other Δfit indices showed desirable values, and the fit even increased with more restricted models. We could, therefore, consider the model invariant between education levels on the configural, metric, scalar, factor mean, and residual variance levels. Measurement invariance between the second (*n* = 369) and sixth weeks (*n* = 273) was reached on the configural level. Even though the χ^2^-test was significant on both the metric and scalar invariance levels, other Δfit indices showed desirable values, and the fit even increased with more restricted models. We could, therefore, consider the model invariant in time on the configural, metric, scalar, and factor mean levels. The final model was non-invariant only on the level of residual variances between the second and sixth weeks of measurement.

**TABLE 6:** Measurement invariance for Model 4 across age, gender, and education level.

	**χ^2^(df)**	**TLI**	**RMSEA**	**SRMR**	**Δχ^2^**	**Δdf**	**ΔTLI**	**ΔRMSEA**	**ΔSRMR**
**Age**									
***Configural***	**58.11 (26)**	0.988	0.083	0.042	–	–	–	–	–
***Metric*** *(loadings, free var, free means)*	65.18 (31)	0.989	0.078	**0.042**	5.76	5	0.001	–0.005	0.00
***Scalar*** *(loadings, intercepts, free var, free means)*	79.00 (36)	0.988	0.082	0.043	11.92*	5	–0.001	0.003	0.00
***Factor means***	61.05 (38)	**0.994**	**0.058**	0.043	0.48	2	0.006	–0.023	0.00
***Residuals***	101.3 (43)	0.987	0.087	0.052	18.97**	7	–0.002	0.005	0.01
**Gender**									
***Configural***	**52.60 (26)**	0.991	0.075	**0.041**	–	–	–	–	–
***Metric*** *(loadings, free var, free means)*	58.77 (31)	0.992	0.071	0.041	5.65	5	0.001	–0.005	0.000
***Scalar*** *(loadings, intercepts, free var, free means)*	65.51 (36)	0.992	0.067	0.041	6.58	5	0.001	–0.003	0.000
***Factor means***	59.98 (38)	**0.995**	**0.057**	0.041	2.11	2	0.002	–0.011	0.000
***Residuals***	90.90 (43)	0.990	0.079	0.055	16.74*	7	–0.003	0.011	0.014
**Education**									
***Configural***	**64.19 (26)**	0.985	0.091	**0.046**	–	–	–	–	–
***Metric*** *(loadings, free var, free means)*	76.73 (31)	0.985	0.091	0.047	11.54*	5	0.000	0.000	0.001
***Scalar*** *(loadings, intercepts, free var, free means)*	88.38 (36)	0.985	0.090	0.047	10.52*	5	0.000	–0.001	0.001
***Factor means***	76.33 (38)	**0.990**	**0.075**	0.047	2.35	2	0.005	–0.015	0.000
***Residuals***	96.26 (43)	0.987	0.083	0.056	11.71	7	0.002	–0.007	0.009
**Time** (comparing week 2 and week 6)									
***Configural***	**66.49 (26)**	0.994	0.070	**0.029**	–	–	–	–	–
***Metric*** *(loadings, free var, free means)*	71.35 (31)	0.995	0.064	0.029	3.18	5	0.001	–0.006	0.000
***Scalar*** *(loadings, intercepts, free var, free means)*	85.44 (36)	0.995	0.066	0.029	11.84*	5	0.000	0.002	0.000
***Factor means***	71.66 (38)	**0.997**	**0.053**	0.029	2.88	2	0.002	–0.013	0.000
***Residuals***	120.7 (43)	0.993	0.075	0.037	25.92***	7	–0.002	0.010	0.007

*p < 0.05, **p < 0.01, ***p < 0.001. Values in bold are the best fitting values when all invariance models within the same groups are compared. The factor mean level and residual level were both compared with the scalar level.

### Reliability and Convergent Validity

The internal consistency of the final model was ω = 0.86 for the affective and ω = 0.81 for the behavioral domains (see Table 5). Additionally, the internal consistency of the general factor in model 1 was ω = 0.91. None of the GCS items would increase the internal consistency when dropped.

Thirteen participants had missing data on the GSRS scale, resulting in 367 patients. With respect to the final two-factor model with affective and behavioral dimensions (model 4), the affective domain was correlated more strongly with the GSRS (*r* = 0.449, *p* < 0.05) than the behavioral domain was (*r* = 0.290, *p* < 0.05). Additionally, a small to moderate positive correlation between the latent constructs of the unidimensional GCS (Model 1) and GSRS scales was found (*r* = 0.394, *p* < 0.05).

## Discussion

The present study described the validation of the Czech version of the Group Cohesiveness Scale (GCS). The average item scores and reliability were compatible with those of the original Thai version (Wongpakaran et al., 2013). However, we concluded that, based on a CFA, the most preferable model was a two-factor solution with the correlated affective and behavioral domains (model 4). This solution is more parsimonious than the fine-tuned unidimensional solution (model 2) suggested by Wongpakaran et al. (2013).

The final model demonstrated excellent fit and was invariant across age groups, genders, education levels, and time. The Czech version did not even show any problematic functioning of item 6 as presented in the English translation by Poyner-Del Vento et al. (2018). Theoretically, group cohesion is related to the working alliance (Johnson et al., 2005). However, in our study, we found only small to medium correlations between the GCS subscales and the GSRS. This finding was unexpected, since the GSRS measures patients’ relationships not only with the therapists/group leaders but also with other members of the group; therefore, there is an apparent overlap in what the instrument is expected to measure. Although the affective domain was more promising than the behavioral domain in terms of convergent validity, overall, the convergent validity of the GCS was not particularly supported in this study.

### Theoretical Support for Two-Dimensional Group Cohesion

The GCS was conceived as a unidimensional construct by Wongpakaran et al. (2013). However, the unidimensional model (model 1) demonstrated an acceptable fit neither in their study nor in ours. Although the large correlation between the affective and behavioral factors may be interpreted in favor of the unidimensionality of the scale, the two dimensions are still independent to some degree and represent different phenomena conceptually. Theoretical support for the two-factor model with the affective and behavioral domains can already be found in the standardization study by Wongpakaran et al. (2013), even though these authors did not report fit indices for this model. Group cohesiveness has been recognized as a multidimensional construct several times in the past (Hogg, 1993). Mudrack’s (1989) definition of group cohesion as a combination of attraction-to-group and commitment to the group task provides a solid rationale for the differentiation of group cohesion into the affective and behavioral domain. The former is associated with the attraction to the group or its members and by collectively sharing positive, as well as negative, emotional experiences (Barsade and Knight, 2015). The latter, on the other hand, is associated with a commitment to the group (Mudrack, 1989) that may be manifested, for instance, by following group rules or giving gifts to other members (Lawler et al., 2000). Another literature supporting the two-dimensional model was Carron et al. (1985) who defined the individual group factor (commitment to other members of group) and task-social factor (interest in the goals of the group). Cota et al. (1995) in their review of group cohesion structure discussed both unidimensionality and multidimensionality resulting in favoring the multidimensional perspective (normative and behavioral components are divided and considered primary components of group cohesion). Kipnes et al. (2002) tested group cohesion dimensionality using two different instruments and claimed that cohesion is a multidimensional construct and offer a hierarchical structure [first order factors will be (1) bond to individual members and (2) level of trust and encouragement of the group as a whole].

In summary, given the high internal consistency of the unidimensional solution and the large correlation between the affective and behavioral dimensions, the GCS may be used as an essentially unidimensional measure of group cohesiveness. However, it should be done with caution and with the awareness of the fact that group cohesiveness may be, in fact, composed of different and partially independent phenomena.

### Similarities and Differences Between the Thai and Czech Versions

The Czech version of the GCS demonstrated some features similar to those of the Thai version. Both versions were characterized by similar values of item-total correlations and internal consistency. Item loadings in terms of the unidimensional model were very similar for both versions as well. The GCS scores were relatively skewed in both studies. Patients tended to perceive their groups as rather cohesive in both cultures. Based on these similarities, we can argue that both versions are comparable.

However, certain differences between the Czech and Thai versions can be found. The two-dimensional solution as the best fitting solution is different from the original unidimensional solution. This may be attributed to cultural differences. Furthermore, the mean total score of the unidimensional model was higher in the Thai version (4.7) than in the Czech version (3.7). Therefore, Thai participants might perceive therapeutic groups as generally more cohesive than Czech participants do or might be less willing to report a lack of cohesion.

### Limitations

First, the sample was relatively heterogeneous and did not represent both genders equally (70% were female). Although this corresponds to the fact that most psychotherapy clients are women, future studies may investigate male groups to explore possible differences in the factor structure of group cohesion. Second, 67 patients dropped out of the study by the second week (i.e., the time when the first measurement of group cohesion took place). Although there were no significant differences between those who dropped out and those who continued with the treatment, this number of participants could have changed some subtle structures within the data. Third, two models yielded a satisfactory fit. The selection of the final model, even though theoretically anchored, is always relatively arbitrary in such cases. Moreover, none of the models fulfilled the criteria for a good fit regarding the χ^2^/df fit index. However, the chi-square test of model fit (and its derivatives) are sample size sensitive and could lead to the rejection of factor model even when residual variances are negligible. Fourth, the final two-factor model was invariant across age cohorts, genders, education levels, and time. Nevertheless, response options 1, 2, and 3 were clustered into a single response option because of missing response patterns in the data. This reduction of thresholds might have distorted our conclusions about the invariance. This response pattern might be explained by the tendency of group members to perceive their group likewise; hence, their responses to the measurement tool or to particular items could be limited to a very homogenous response style (Evans and Jarvis, 1980).

## Conclusion

The Czech version of the GCS is a reliable and psychometrically valid tool for the measurement of the affective and behavioral domains of group cohesiveness. Thanks to its brevity, the scale is useful in the rapid hospital or therapeutic environment. As far as we know, this is the first psychometric validation of the GCS in Western culture and the Caucasian population. In this study, we revised the originally proposed unidimensional factor structure (Wongpakaran et al., 2013) and found support for the existence of the affective and behavioral domain of group cohesion.

## Data Availability Statement

The data analyzed in this study is subject to the following licenses/restrictions: The datasets analyzed during the current study are available from the corresponding author on reasonable request. Requests to access these datasets should be directed to AK, klocek.adam@mail.muni.cz; https://www.researchgate.net/publication/344571321_GCS_full_dataset.

## Ethics Statement

The studies involving human participants were reviewed and approved by the Research Ethics Committee of Masaryk University (Ref. No. EKV-2017-029-R1). The patients/participants provided their written informed consent to participate in this study.

## Author Contributions

AK: conceptualization, theoretical literature search, analysis, writing, and reviewing. TŘ: conceptualization and reviewing. HC: analysis and reviewing. All authors contributed to the article and approved the submitted version.

## Conflict of Interest

The authors declare that the research was conducted in the absence of any commercial or financial relationships that could be construed as a potential conflict of interest.

## Footnotes

1 The GCS used in this study is unrelated to the Harvard Community Health Plan Group Cohesiveness Scale (which is also referred to as GCS in the literature; Budman et al., 1993).

2 The supplemental therapeutic techniques and session differed by site.

3 One site was characterized by the session length of 75 min.
